# Supplementary material for: 2-Oxonanonoidal Antibiotic Actinolactomycin Inhibits Cancer Progression by Suppressing HIF-1α
Source: Cells. 2019 May 10;8(5):439. doi: 10.3390/cells8050439 (PMC6563139; doi:10.3390/cells8050439)
Supplement: Supplementary file 1 [file cells-08-00439-s001.pdf]

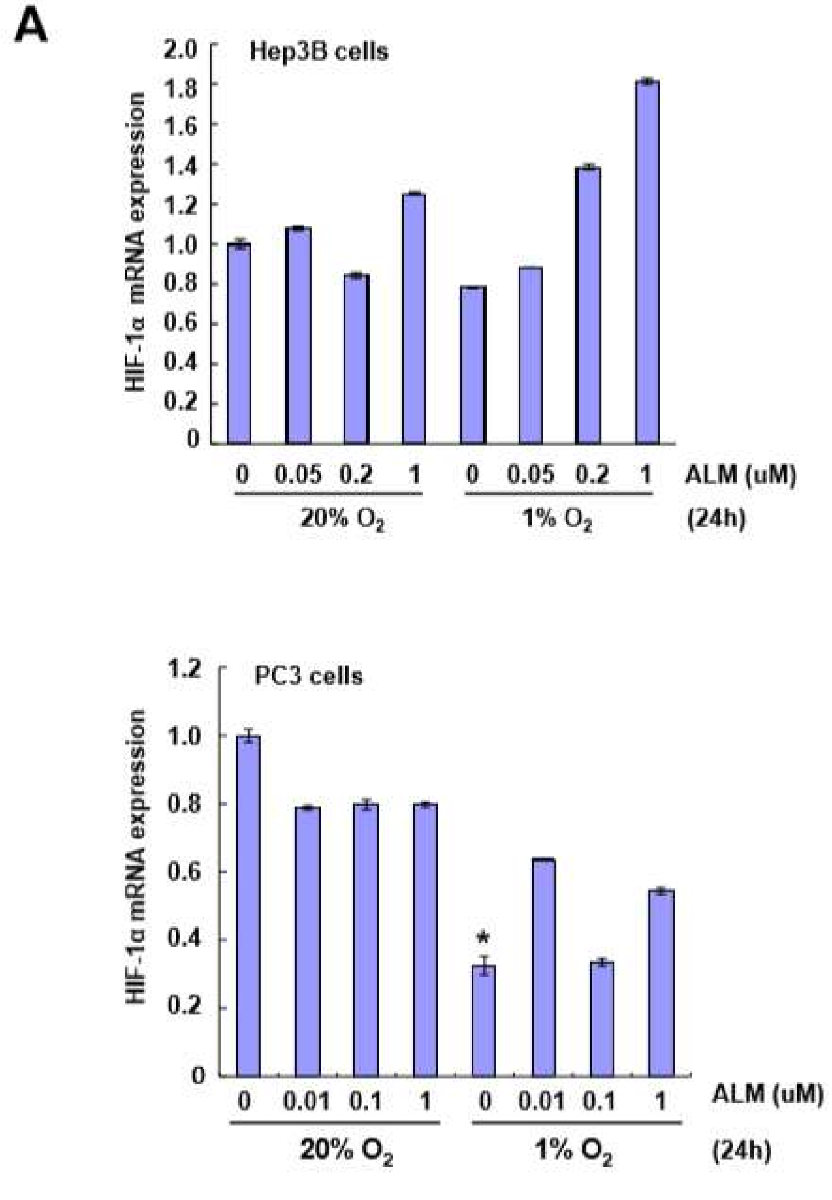

Supplementary Figure 1. ALM has no effect on HIF-1α mRNA expression

(A) Hep3B and PC3 cells were exposed to vehicle or the indicated concentration of ALM for 24 hours under normoxic or hypoxic conditions and total RNA was subjected to RT-PCR assays for HIF-1α. *$P < 0.05$ compared to normoxic control group.

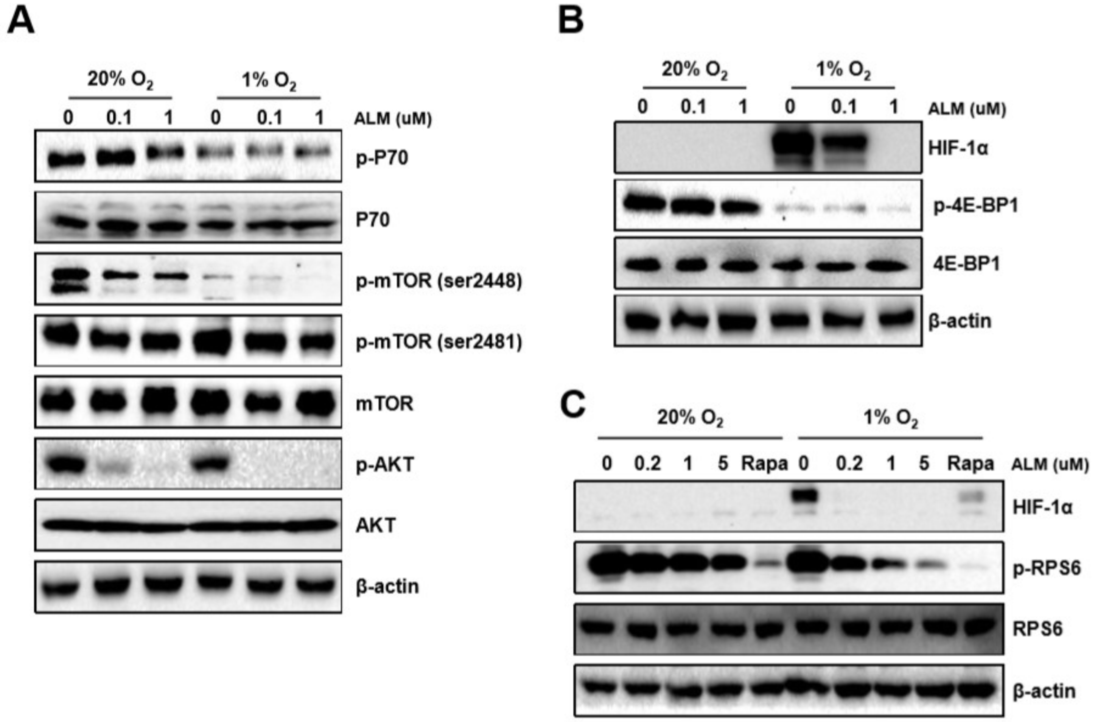

Supplementary Figure 2. ALM inhibits HIF-1α translation by down-regulating mTOR pathway

(A-C) Hep3B cells were cultured at 20% or 1% O_2 for 24 hours in the presence of ALM (at the indicated concentrations), rapamycin or not, and whole cell lysates were subjected to for: (A) p-P70, p-Akt, p-mTOR (2448/2481), mTOR, (B) p-4E-BP1, (C) p-RPS6, HIF-1α or β-actin.

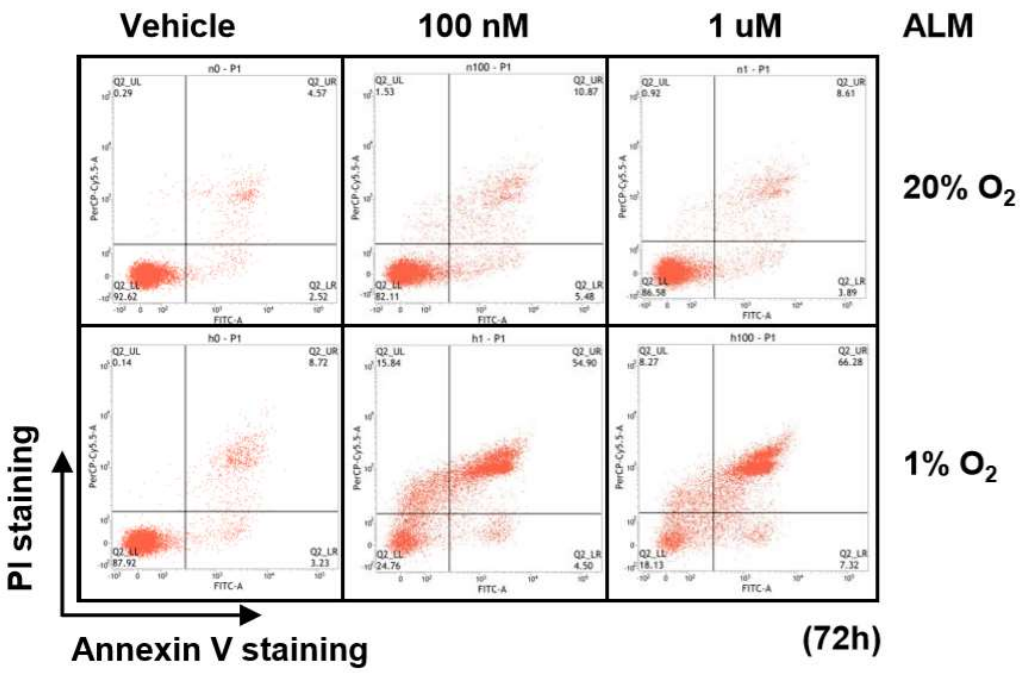

Supplementary Figure 3. ALM induces cell apoptosis in PC3 cells. PC3 cells were treated with indicated dose and time of ALM under normoxia/hypoxia and apoptosis population was analyzed by protocol described in Figure 3D.

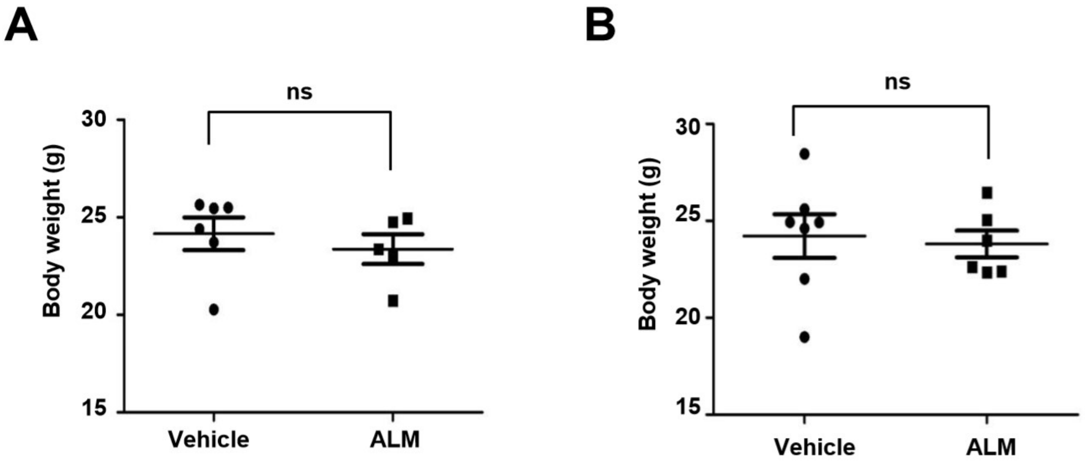

Supplementary Figure 4. Effects of ALM on mouse body weight. (A, B) Body weight were weighted after sacrificing mice (mean ± s.e.m., P value is determined by Student's t-test).

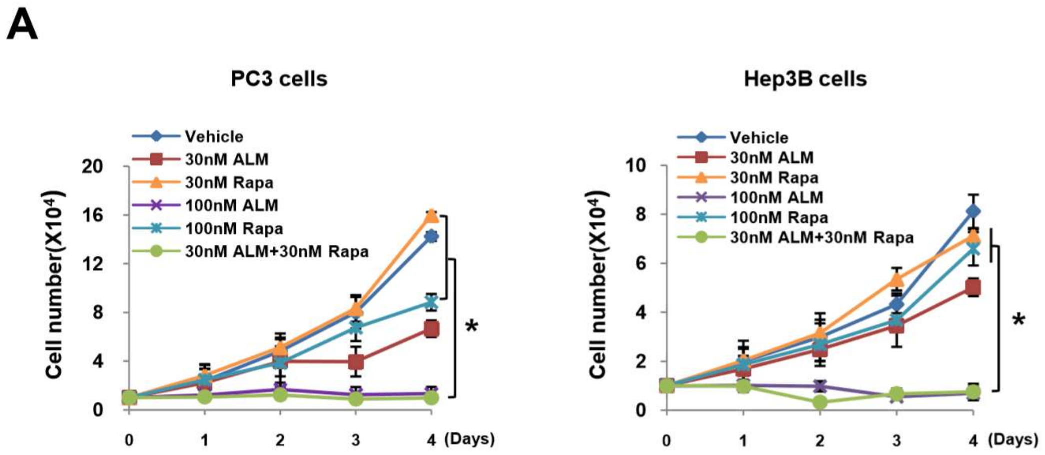

Supplementary Figure 5. Low dose of ALM enhances the inhibitory effect of mTOR inhibitor rapamycin on cell growth

(A) Cell growth curves of PC3 and Hep3B cells treated with indicated dose of vehicle, ALM, rapamycin, or combination of ALM with rapamycin. *$P < 0.05$ compared between the indicated groups.

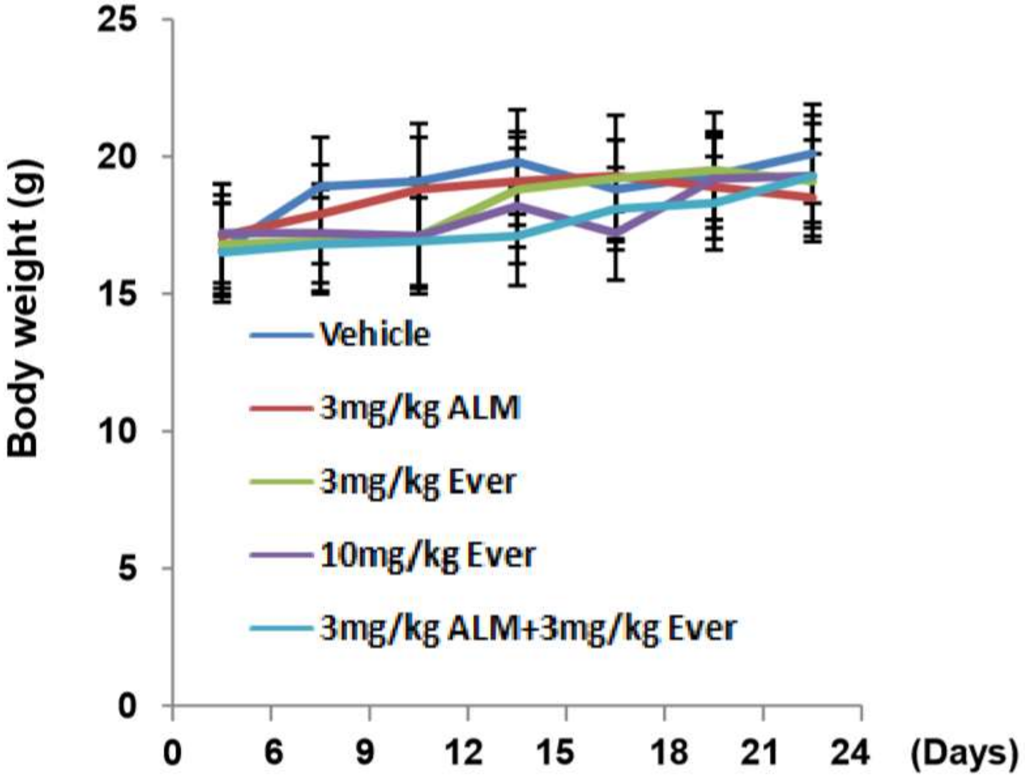

Supplementary Figure 6. Effects of ALM and mTOR inhibitor on mouse body weight.

Body weight was determined from day 6 to day 24.

Supplementary Table 1. Nucleotide sequences of primers used for qRT-PCR

primer	Forward Sequence (5' - 3')	Reverse Sequence (5' - 3')
HIF-1α	CCACAGGACAGTACAGGATG	TCAAGTCGTGCTGAATAATACC
Bnip3	AACTCAGATTGGATATGGGATTGG	AGAGCAGCAGAGATGGAAGG
HK1	TGGAGTCCGAGGTTTATG	TTTGGATTGTTGGCAAGG
18S	CGGCGACGACCCATTCGAAC	GAATCGAACCCTGATTCCCCGTC
